# Meetings of Societies

**Published:** 1938

**Authors:** 


### Meetings of Societies

### Bristol Medico-Chirurgical Society

13th April.

Dr. T. M. Ling: "**The Emotional Factor in Disease.**" (Page 101.)

11th May.

Mr. F. W. Willway: "**Problems in the Diagnosis and Treatment of Intracranial Lesions.**"

